# The Effect of the Interaction of Nitrogen Fertilization with Planting Density on Maize (*Zea mays* L.) Yield, Stalk Mechanical Properties, and Enzyme Activity

**DOI:** 10.3390/plants15030459

**Published:** 2026-02-02

**Authors:** Pei Chen, Li Zhao, Zhi-Long Zhang, Lin-Zhuan Song, Xue-Feng Zhao, Xin Zhang, Xin-Rong Duan, Min Liang, Chang Zhang, Chuang-Yun Wang

**Affiliations:** 1 College of Agriculture, Shanxi Agricultural University, Jinzhong 030801, China; 20233239@stu.sxau.edu.cn (P.C.); lizhao@sxau.edu.cn (L.Z.); 15947185502@163.com (Z.-L.Z.); 202430209@stu.sxau.edu.cn (L.-Z.S.); 202420172@stu.sxau.edu.cn (X.Z.); 202430127@stu.sxau.edu.cn (X.-R.D.); 20233243@stu.sxau.edu.cn (M.L.); 20232189@stu.sxau.edu.cn (C.Z.); 2 Yuci District Meteorological Bureau of Shanxi Province, Jinzhong 030600, China; ertou01@163.com

**Keywords:** nitrogen management, group configuration, mechanical properties, physiological enzyme activity, yield

## Abstract

This study examines the effects of nitrogen-planting density interactions on stalk lodging resistance mechanisms and yield formation in spring maize (*Zea mays* L.), aiming to establish a theoretical framework for optimizing planting configurations to achieve high and stable yields in Shanxi Province. Using the maize variety Qiangsheng 192 as the experimental material, a split-plot field experiment was conducted from 2023 to 2024. Planting density served as the main plot, with three levels: 60,000 plants ha^−1^ (M1, 6 plants m^−2^, control), 75,000 plants ha^−1^ (M2, 7.5 plants m^−2^), and 90,000 plants ha^−1^ (M3, 9 plants m^−2^), each replicated three times. Nitrogen application rate was the subplot, with four treatments: N0 (0 kg ha^−1^), N1 (90 kg ha^−1^), N2 (180 kg ha^−1^), and N3 (270 kg ha^−1^). At the tasseling stage, agronomic traits and mechanical properties of the stalks were investigated. The activities of Phenylalanine Ammonia-Lyase (PAL), Tyrosine Ammonia-Lyase (TAL), and Cinnamyl Alcohol Dehydrogenase (CAD) in the stalks were measured at the big trumpet stage, tasseling stage, filling stage, and maturity stages, and yield was determined. The results showed that the M2 treatment achieved the highest yield, followed by M3, while M1 (control) had the lowest yield. Under the M2N2 configuration, the yield reached 13.55 Mg ha^−1^, the highest recorded. As planting density increased, maize growth exhibited variations: the basal internodes elongated, mechanical properties declined, and the activities of PAL, TAL, and CAD enzymes decreased. Increased nitrogen application improved basal internode quality. Correlation analysis revealed that stalk mechanical properties were positively correlated with PAL, TAL, and CAD enzyme activities, which could both reflect the quality of the stalk. In conclusion, the M2N2 configuration is an optimal combination for enhancing maize yield, improving stalk mechanical properties, and increasing enzyme activity, making it suitable for large-scale application in the dryland spring maize areas of Shanxi Province.

## 1. Introduction

Maize (*Zea mays* L.), as a crucial food crop in China, plays a central role in the stability and development of the national economy [1]. The study by Luo et al. [2] revealed that in the major maize-growing regions of China, a significant increase in grain yields, ranging from 13% to 20.8%, was achieved by finely adjusting planting density. Compared to simply expanding the planting area, increasing planting density can effectively increase the number of plants per unit area [3], thereby becoming a key strategy to enhance maize yields [4]. However, the increase in yield brought about by dense planting is not without cost, and it is accompanied by an increase in lodging rate and lodging grade [5,6,7]. With the rise in planting density, the competition for resources among maize groups becomes increasingly intense, resulting in stunted individual development and elongated stalks, which further weaken the compressive strength and rind puncture strength of the stalks [8,9]. Although the activity of enzymes related to lignin synthesis increases at the initial stage of growing planting density, excessive planting density will inhibit this process [10]. Varieties with high dry matter accumulation and lignin content in the stalk exhibit stronger resistance to lodging [11]. Lignin, a complex phenolic heteropolymer, is the most important secondary metabolite produced in plant cells through the phenylalanine/tyrosine metabolic pathway [12], and Cinnamyl Alcohol Dehydrogenase is the final step in the lignin synthesis reaction. Phenylalanine Aminolyase-lyase, Tyrosine Ammonia-lyase, and Cinnamyl Alcohol Dehydrogenase play crucial roles in plant stress response [13], and their activity levels reflect the lignin content. Phenylalanine Aminolyase-lyase is the rate-limiting enzyme in the shikimate metabolic pathway, catalyzing the conversion of L-phenylalanine to trans-cinnamic acid. At the same time, Cinnamyl Alcohol Dehydrogenase can reduce cinnamyl alcohols to corresponding lignin monomers, and Tyrosine Ammonia-lyase catalyzes the production of coumaric acid from tyrosine, which is unique to gramineous plants [14].

Nitrogen, as an indispensable nutrient element for crop growth, is often referred to as the “source of life” for food crops. Nearly half of the world’s population relies on fertilizers for food production, with nitrogen fertilizers accounting for 30% to 50% of this reliance [15]. Lu et al. [16] noted that an optimal amount of nitrogen fertilizer application can enhance the agronomic traits of maize, thereby improving its resistance to lodging. The research of Gao et al. [17] and Liu et al. [9] further confirmed that, with an increase in planting density, the mechanical properties of stalks, such as compressive strength and rind puncture strength, gradually weaken. In contrast, the accumulation of dry matter in stalks can be effectively improved by increasing the nitrogen application rate, thereby enhancing the mechanical strength of the stalks and improving lodging resistance [18,19]. However, over-application of nitrogen fertilizer is counterproductive, resulting in a decrease in stalk strength and an increased risk of lodging. Liu et al. [20] demonstrated that with the increase in nitrogen fertilizer, the lignin-related enzyme activity, lignin, cellulose, and hemicellulose contents all exhibited a downward trend. The experimental results of Zhan et al. [21] demonstrated that the application of appropriate nitrogen fertilizer can increase lignin content. In contrast, excessive nitrogen fertilizer significantly increased plant height and ear height of maize, markedly elongated internode length, and significantly reduced stalk-breaking resistance, thereby increasing the risk of maize lodging. Therefore, the rational application of nitrogen fertilizer has dual significance: it enables high yield and efficiency in maize production while also promoting environmental protection.

Currently, most studies primarily focus on the individual effects of either planting density or nitrogen application on the mechanical properties, enzymatic activities, and yield of maize stalks [8,22,23]. In contrast, research on the interactive effects of nitrogen fertilization and planting density remains relatively limited. This study aims to investigate the influence of these two factors, planting density and nitrogen application rate, on the agronomic traits of maize stalks, the mechanical properties and enzymatic activities of basal internodes, as well as yield-related traits. By elucidating the influence of planting density and nitrogen application on maize growth, this research seeks to provide practical management strategies and theoretical support for enhancing maize yield in Shanxi Province.

The following hypotheses were proposed: (1) The interaction between nitrogen-planting density affected stalk quality by changing the population structure (light and ventilation), thereby regulating yield. (2) The appropriate combination of planting density and nitrogen fertilizer can enhance the activity of enzymes related to lignin synthesis and improve lodging resistance. (3) Excessive planting density intensifies resource competition, leading to yield penalties, while inappropriate nitrogen application rates promote excessive vegetative growth, resulting in tall but structurally weak plants prone to lodging. The results of this study are expected to provide a theoretical basis and technical framework for the synergistic adaptation of “nitrogen-planting density” in regional high-yield and high-efficiency maize cultivation.

## 2. Materials and Methods

### 2.1. Experimental Materials

The drought-tolerant maize (*Zea mays* L.) cultivar ‘Qiangsheng 192’ (supplied by Fushengyuan Science and Technology Development Co., Ltd., Taiyuan, Shanxi, China) was used in this study. This maize variety is a hybrid used for grain production, with a growth period of approximately 128 days. The plant architecture is semi-compact, the ear is conical to cylindrical, the cob is red, the kernel is yellow, and it is a dent type with strong resistance to lodging, high resistance to *Stalk Rot*, moderate resistance to *Ear Rot*, more sensitive to *Silky Spike Disease*, *Great Spot Disease* and *Dwarf Foliage Disease*.

### 2.2. Growth Conditions

**Figure 1:**
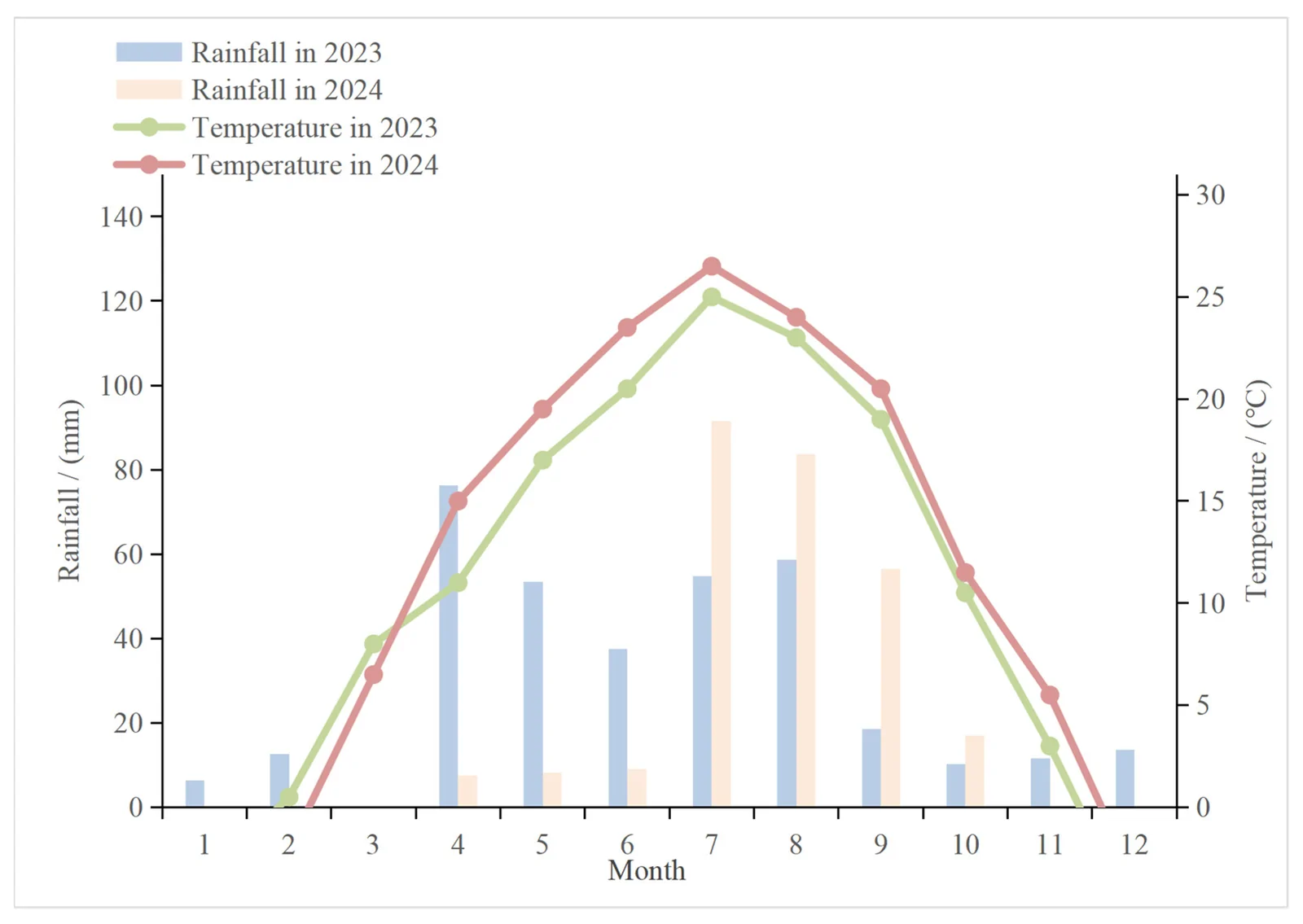
Rainfall and temperature changes in the study area from 2023 to 2024.

The experiment was conducted from 2023 to 2024 at the Dongyang Experimental Base of Shanxi Agricultural University, Yuci District, Jinzhong City, Shanxi Province (112°40′34″ E, 37°32′55″ N). All data in this experiment are the average of two years of data, and the two-year reproductive period is shown in Table 1. The region is characterized by a warm-temperate, sub-humid, continental monsoon climate [24]. Rain and heat occurred in the same season. The average annual rainfall from 2023 to 2024 was 410.85 mm, with an average monthly temperature of 11.27 °C. During the reproductive period, precipitation levels were 222.9 mm in 2023 and 248.9 mm in 2024, with the majority of rainfall concentrated between July and September (Figure 1). The soil in the experiment site was cinnamon soil with an organic matter content of 14.85 g kg^−1^, total nitrogen content of 0.59 g kg^−1^, alkali-hydrolyzed nitrogen content of 56.65 mg kg^−1^, total phosphorus content of 1.20 g kg^−1^, available phosphorus content of 28.79 mg kg^−1^, and total potassium content of 21.94 g kg^−1^. The content of available potassium was 274.54 mg kg^−1^, with a pH of 8.26.

### 2.3. Experimental Design and Field Management

The experiments employed a split-plot design with three replicates. Planting density served as the main plot factor, and nitrogen application rate as the subplot factor. Three plant densities were used, including 6 × 104 plants ha^−1^ (M1) (local conventional planting density), 7.5 × 104 plants ha^−1^ (M2), and 9 × 104 plants ha^−1^ (M3). Four nitrogen levels were included as subplots, which were 0 kg ha^−1^ (N0), 90 kg ha^−1^ (N1), 180 kg ha^−1^ (N2), and 270 kg ha^−1^ (N3). The plots were 40 m^2^ with a row spacing of 0.5 m. Nitrogen fertilizer (Urea: 46% N) was applied at the three-leaf stage of maize. Weed management was conducted through pre-emergence herbicide application using a soil-sealing herbicide mixture within three days of sowing, consisting of 40% atrazine suspension concentrate (SC) at 3000 mL ha^−1^ and 90% acetochlor emulsifiable concentrate (EC) at 1500 mL ha^−1^, followed by manual weeding during the crop growth period. For pest and disease management, aphids were controlled at the seedling stage using 10% imidacloprid WP at 150 g ha^−1^. Corn borers (*Ostrinia furnacalis*) were managed at the large trumpet stage by spraying 3% methyl parathion. Additionally, 50% carbendazim WP at 900 g ha^−1^ was applied at the early onset of symptoms to prevent northern corn leaf blight (*Exserohilum turcicum*).

### 2.4. Sampling and Measurements

#### 2.4.1. Stalk Agronomic Traits

At the tasseling stage, ten maize plants with uniform growth were randomly selected from the middle row of each plot. The plant height (cm), ear height (cm), and center of gravity height (cm) of the maize were measured in the field with a tower ruler, and the ear height coefficient (%) [21] was calculated. The internode lengths of the 3rd, 4th, and 5th internodes at the base of stalks were measured with a straightedge and a vernier caliper (cm). The diameters of the long and short axes in the middle of the internodes were measured (mm), and the cross-sectional area of the internode (mm^2^) was calculated [8].

Calculations: (1) Ear height coefficient (%) = spike height/plant height.

(2) Cross-sectional area of internode (mm^2^) = 1/4 × π × L1 × L2, where L1 is the long axis diameter and L2 is the short axis diameter.

#### 2.4.2. Stalk Mechanical Properties

At the tasseling stage, nine uniformly growing maize plants (with plant height, leaf age, and stalk diameter close to the population average) were randomly selected from the central rows of each plot. Plants showing signs of disease, insect damage, chlorosis, malformed leaves, mechanical injury, or significantly delayed or advanced growth were excluded. After removing leaf sheaths and roots, the 3rd, 4th, and 5th basal internodes of each stalk were isolated. The stalks were grouped into sets of three, and the rind penetration strength, compressive strength, and bending strength of the internodes were measured in the field using a YYD-1 maize stalk strength tester (Zhejiang Tuoyi Co., Ltd., Yongkang, China). Each measurement was repeated three times, and the average value was calculated.

(1) Stalk rind puncture strength: A probe with a cross-sectional area of 0.01 cm^2^ was inserted perpendicularly into the midpoint of the internode at a uniform slow speed until the stem ruptured. The value was then recorded.
(2) Stalk compressive strength: Each internode was placed horizontally in the groove of the tester and compressed rapidly until failure. The maximum force was recorded.
(3) Stalk bending strength: The basal section of the stalk was placed horizontally in the groove of the tester and pressed rapidly until the stalk was crushed. The corresponding value was recorded.

#### 2.4.3. Stalk Stem Enzyme Activity

At the big trumpet stage, tasseling stage, filling stage, and maturity stages, three uniformly growing plants were randomly selected from the middle rows of each treatment, avoiding border rows (such as plant height, leaf age, and stem diameter, which should approximate the average values of the population). Additionally, plants with pests, diseases, yellowing or deformed leaves, mechanical damage, or significantly delayed or accelerated growth were excluded from the study. The basal 3rd internode of each plant was excised, immediately placed in foam boxes containing ice packs, and transported to the laboratory for storage at −80 °C. For enzymatic assays, a 3–4 cm segment from the middle portion of the 3rd internode was ground in liquid nitrogen. The activities of Phenylalanine Ammonia-lyase (PAL), Tyrosine Ammonia-lyase (TAL), and Cinnamyl Alcohol Dehydrogenase (CAD) were determined using commercial assay kits (purchased from Bioleaper Biotechnology Co., Ltd., Shanghai, China; website: www.bioleaper.com), following the manufacturer’s protocols.

1. Determination of Phenylalanine Ammonia-lyase (PAL) activity: The maize stalks were ground in liquid nitrogen, and 0.1 g of the powder was weighed. Then, 1 mL of extraction buffer was added, and the mixture was homogenized in an ice bath. The homogenate was centrifuged at 10,000× *g* for 10 min at 4 °C. The supernatant was collected and kept on ice for subsequent analysis. Subsequently, 5 μL of the supernatant and the corresponding reagents were added to a 96-well UV-transparent microplate according to the order specified in the assay table. The mixture was immediately stirred and incubated at room temperature. The absorbance at 290 nm was recorded after 10 min (A1) along with the absorbance of the control group (A2). The difference in absorbance was calculated as ∆A = A1 − A2.

Phenylalanine Ammonia-lyase enzyme activity calculation:

One unit of enzyme activity was defined as a change of 0.05 in absorbance at 290 nm per minute per gram of fresh tissue per milliliter of the reaction mixture.

PAL activity (U g^−1^ fresh weight) = ∆A × V_total_reaction ÷ (W × V_sample ÷ V_total_extract) ÷ 0.05 ÷ T = 26.6 × ∆A ÷ W.

Note: V_total_reaction: total volume of the reaction system, 0.2 mL. V_sample: volume of sample added, 0.005 mL. V_total_extract: total volume of extraction buffer added, 1 mL. T: reaction time, 30 min. W: sample mass, g.

2. Determination of Tyrosine Ammonia-lyase (TAL) activity: The maize stalks were ground in liquid nitrogen, and 0.1 g of the powder was weighed. Then, 1 mL of extraction buffer was added, and the mixture was homogenized in an ice bath. The homogenate was centrifuged at 8000× *g* for 10 min at 4 °C. According to the sample assay table, 40 μL of the supernatant and the corresponding reagents were sequentially added into an EP tube. The mixture was immediately vortexed and then centrifuged at 10,000× *g* and 4 °C for 5 min. Subsequently, 200 μL of the resulting supernatant was transferred to a 96-well UV-transparent microplate. The absorbance at 333 nm was measured for both the test sample (A1) and the control (A2). The difference in absorbance was calculated as ∆A = A1 − A2.

Calculation of Tyrosine Ammonia-lyase Activity:

One unit of enzyme activity was defined as the amount of enzyme that caused a change of 0.005 in absorbance at 333 nm per minute per gram of fresh tissue per milliliter of the reaction mixture.

TAL activity (U g^−1^ fresh weight) = ∆A × V_total_reaction ÷ (W × V_sample ÷ V_total_extract) ÷ 0.005 ÷ T = 35 × ∆A ÷ W.

Note: V_total_reaction: total volume of the reaction system, 0.42 mL. V_sample: volume of sample added, 0.04 mL. V_total_extract: total volume of extraction buffer added, 1 mL. T: reaction time, 60 min. W: sample mass, g.

3. Determination of Cinnamyl Alcohol Dehydrogenase (CAD) activity: The maize stalks were ground in liquid nitrogen, and 0.1 g of the powder was weighed. Then, 1 mL of extraction buffer was added, and the mixture was homogenized in an ice bath. The homogenate was centrifuged at 8000× *g* for 10 min at 4 °C. The supernatant was collected and kept on ice for subsequent analysis. Subsequently, 10 μL of the supernatant and the corresponding reagents were added to a 96-well microplate in the order specified in the assay table. The mixture was thoroughly mixed, and the initial absorbance at 340 nm (A1) was immediately recorded, followed by a second measurement after 5 min (A2). The difference in absorbance was calculated as ΔA = A2 − A1.

Calculation of Cinnamyl Alcohol Dehydrogenase Activity:

One unit of enzyme activity was defined as the amount of enzyme that produced 1 nmol of NADPH per minute per gram of fresh tissue.

CAD activity (nmol min^−1^ g^−1^ fresh weight) = [∆A × V_total_reaction ÷ (ε × d) × 10^9^] ÷ (W × V_sample ÷ V_total_extract) ÷ T = 1286 × ∆A ÷ W

Note: V_total_reaction: total volume of the reaction system, 2 × 10^−4^ L. ε: molar extinction coefficient of NADPH, 6.22 × 10^3^ L·mol^−1^·cm^−1^. d: optical path length (light path diameter) of the 96-well plate, 0.5 cm. V_sample: volume of sample added, 0.01 mL. V_total_extract: total volume of extraction buffer added, 1 mL. T: reaction time, 5 min. W: sample mass, g.

#### 2.4.4. Field Yield Measurement and Indoor Seed Testing

At harvest, yield was measured from the two center rows of each plot, with the total plant number and actual harvested ear count recorded. Concurrently, 20 randomly selected ears were naturally air-dried. When kernel moisture content fell below 20%, indoor ear characterization was performed to determine ear length (cm), bare top length (cm), and ear grain number. After threshing and air-drying, the 100-grain weight (g) was determined. The ear characterization data were integrated with field yield measurements, and the final grain yield was standardized to 14.0% moisture content.

### 2.5. Statistical Analyses

The experimental data were subjected to analysis of variance (ANOVA) using DPS 15.10 statistical software. When the data met the assumptions of normality and homogeneity of variance for ANOVA, the q-test was employed to compare significant differences (*p* < 0.05) among different treatments. Graphing was performed using Origin 9.65 software to determine the relationship between internode characteristics, factors affecting internode formation, and the mechanical strength of basal internodes. Redundancy analysis was performed using CANOCO 5.0.

## 3. Results Analysis

### 3.1. Effect of Nitrogen-Planting Density Interaction on Agronomic Traits of Maize (Zea mays *L.*) Stalks

Planting density and nitrogen application rate significantly affected plant height, ear height, ear height coefficient, and center of gravity height, and their interaction had a significant impact on plant height and center of gravity height (*p* < 0.05), with a highly significant effect on center of gravity height (*p* < 0.01) (Table 2). In terms of the density effect, compared with M1 (control), the plant heights of M2 and M3 increased by 4.13 cm and 7.83 cm, respectively; ear heights increased by 2.79 cm and 5.25 cm, respectively; and center of gravity heights increased by 1.46 cm and 2.10 cm, respectively. The nitrogen application effect, compared with N0 (control), increased the ear height and center of gravity height of N2 by 6.07% and 3.62%, respectively. N3 exhibited the most significant increases in plant height and ear height, reaching 3.26% and 9.93%, respectively; however, excessive nitrogen application did not further enhance the advantages of lodging-resistant agronomic traits.

### 3.2. Effect of Nitrogen-Planting Density Interaction on the Cross-Sectional Area and Length of Internode of Maize Stalks at the Tasseling Stage

As affected by nitrogen application and planting density, the morphological characteristics of maize stalks showed distinct and regular patterns (Table 3). The basal internode cross-sectional area decreased with an increasing number of internodes (3rd internode > 4th internode > 5th internode), while the internode length increased with a growing number of internodes (3rd internode < 4th internode < 5th internode). Planting density, nitrogen application, and their interaction had significant effects on both traits (*p* < 0.01), whereas nitrogen application alone had no significant impact on the length of the 5th internode. From the perspective of planting density, the 3rd internode cross-sectional areas of M2 and M3 were reduced by 35.71 mm^2^ and 36.82 mm^2^, respectively, compared to M1, while the 3rd internode lengths increased by 0.42 cm and 1.29 cm, respectively. High planting density significantly reduced internode thickness and increased internode length. Analysis of nitrogen levels showed that, compared with N0, N2 resulted in the most significant increase in the 3rd to 5th internode cross-sectional area (14.87% to 17.86%) and the most significant decrease in the 3rd to 5th internode length (8.14% to 10.82%). When N3 was applied, the increase in cross-sectional area stagnated, and the 5th internode length even increased.

### 3.3. Effect of Nitrogen-Planting Density Interaction on Maize Yield and Its Composition

Planting density, nitrogen application rate, and their interaction had significant effects on yield and its constituent indicators (Table 4). Maize yield increased initially but then decreased with increasing planting density. The maximum value was reached under the M2N2 configuration, with a yield of 13.55 Mg ha^−1^. Compared to the local conventional planting density M1 (CK), grain yield increased by 16.38% and 4.90% at planting densities M2 and M3, respectively. The number of grains per spike increased by 21.90 and 8.50 grains in M2 and M3, respectively, compared with M1 (CK). At the same density, yield initially increased and then decreased with increasing nitrogen application rates. N1, N2, and N3 resulted in yield increases of 5.11%, 8.73%, and 2.64%, respectively, compared to N0. Increasing nitrogen fertilizer application reduced the bare top length by 0.18 cm to 0.37 cm.

With increasing nitrogen application rates, ear length, ear grain number, and 100-grain weight also exhibited an initial increase followed by a decrease. These three parameters peaked at the N2 level, having increased by 0.89 cm, 71.50 grains, and 0.85 g, respectively, compared to N0.

### 3.4. Effect of Nitrogen-Planting Density Interaction on Mechanical Characteristics of the Basal Internode of Maize at the Tasseling Stage

#### 3.4.1. Stalk Rind Puncture Strength

**Figure 2:**
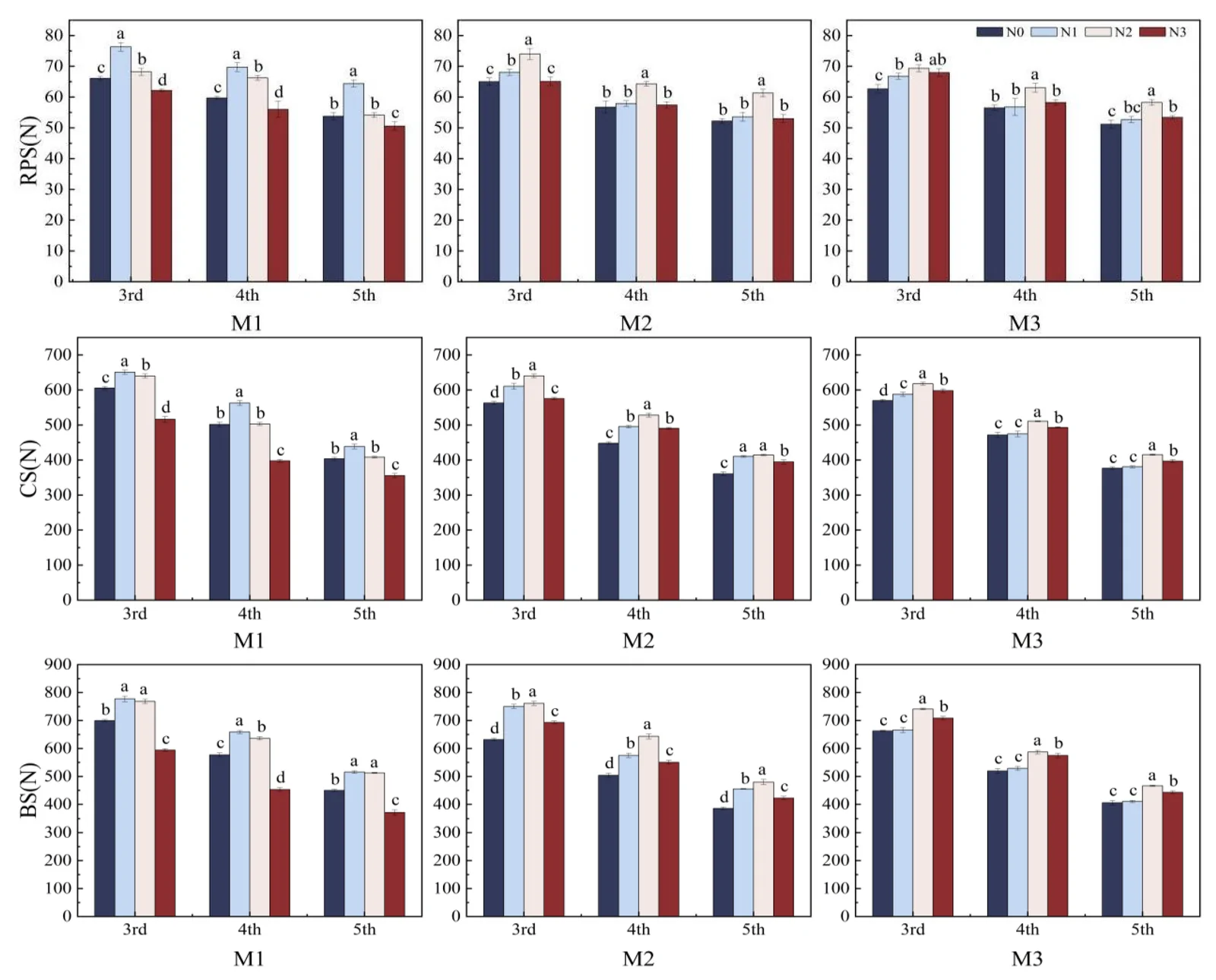
Effects of nitrogen-planting density interaction on the mechanical strength of maize (*Zea mays* L.) stalks. Note: The rind puncture strength (RPS), compressive strength (CS), and bending strength (BS) of maize during the tasseling stage at the 3rd to 5th internodes. M1, M2, and M3 represent planting densities of 60,000, 75,000, and 90,000 plants ha^−1^, respectively; N0, N1, N2, and N3 represent nitrogen application rates of 0, 90, 180, and 270 kg ha^−1^, respectively; “3rd” refers to the 3rd basal internode, “4th” refers to the 4th basal internode, and “5th” refers to the 5th basal internode. a, b, c, and d indicate homogeneous groupings based on the results of the ANOVA. Error bar: represents the standard error.

During the tasseling stage, the rind penetration strength (RPS) of maize stalks decreased gradually with ascending internode position (3rd > 4th > 5th) (Figure 2. RPS). Under the same nitrogen application rate, the RPS decreased with increasing planting density. Compared with the M1 (CK) treatment, the average RPS of maize stalks between the 3rd and 5th internodes decreased by 0.26~6.12% under the M2 treatment and by 2.22~6.81% under the M3 treatment. The ranking of RPS under different nitrogen–density configurations varied: it was N1 > N2 > N0 > N3 for M1, N2 > N1 > N3 > N0 for M2, and N2 > N3 > N1 > N0 for M3. Specifically, compared to N0, the N1 and N2 treatments increased the average RPS by 3.83–5.81 N mm^−2^ and 5.53–6.86 N mm^−2^, respectively. In contrast, the N3 treatment showed a much smaller maximum increase of only 0.48 N mm^−2^.

#### 3.4.2. Stalk Compressive Strength

According to Figure 2 (CS), the compressive strength of maize stalks during the tasseling stage decreased with ascending internode position (3rd > 4th > 5th) and was significantly affected by both planting density and nitrogen fertilizer. Regarding planting density, compared to the M1 (control) treatment, the average CS of the 5th internode showed the greatest reduction under the M2 and M3 treatments, decreasing by 1.58% and 2.28%, respectively. Under different nitrogen fertilizer regimes, the order of compressive strength from the 3rd to 5th internodes during tasseling in the M1 (CK) treatment was N1 > N2 > N0 > N3; in the M2 treatment, the order was N2 > N1 > N3 > N0; and in the M3 treatment, the order was N2 > N3 > N1 > N0. Compared with the N0 level, the average compressive strength of the 3rd internode increased by 36.93 N mm^−2^ at the N1 level, by 53.18 N mm^−2^ at the N2 level, and decreased by 16.21 N mm^−2^ at the N3 level.

#### 3.4.3. Stalk Bending Strength

Figure 2 shows that the bending strength (BS) of maize stalks during the tasseling stage decreased progressively from the 3rd to the 5th internode. Higher planting density reduced BS compared to the control (M1). For the M2 treatment, the average BS of the 3rd, 4th, and 5th internodes decreased by 0.11%, 2.27%, and 5.74%, respectively. The reductions were greater under M3, at 2.16%, 4.94%, and 6.69%, respectively.

Nitrogen application generally increased BS. Compared to N0, the N1 treatment increased the average BS across the 3rd to 5th internodes by 46.70 to 66.39 N mm^−2^. A greater increase (72.63 to 92.17 N mm^−2^) was observed under N2. In contrast, the N3 treatment resulted in only a marginal maximum increase of 0.52 N mm^−2^.

### 3.5. Effect of Nitrogen-Planting Density Interaction on Physiological Characteristics of Maize Basal Internodes

#### 3.5.1. Effect of Nitrogen-Planting Density Interaction on Phenylalanine Ammonia-Lyase Activity

**Figure 3:**
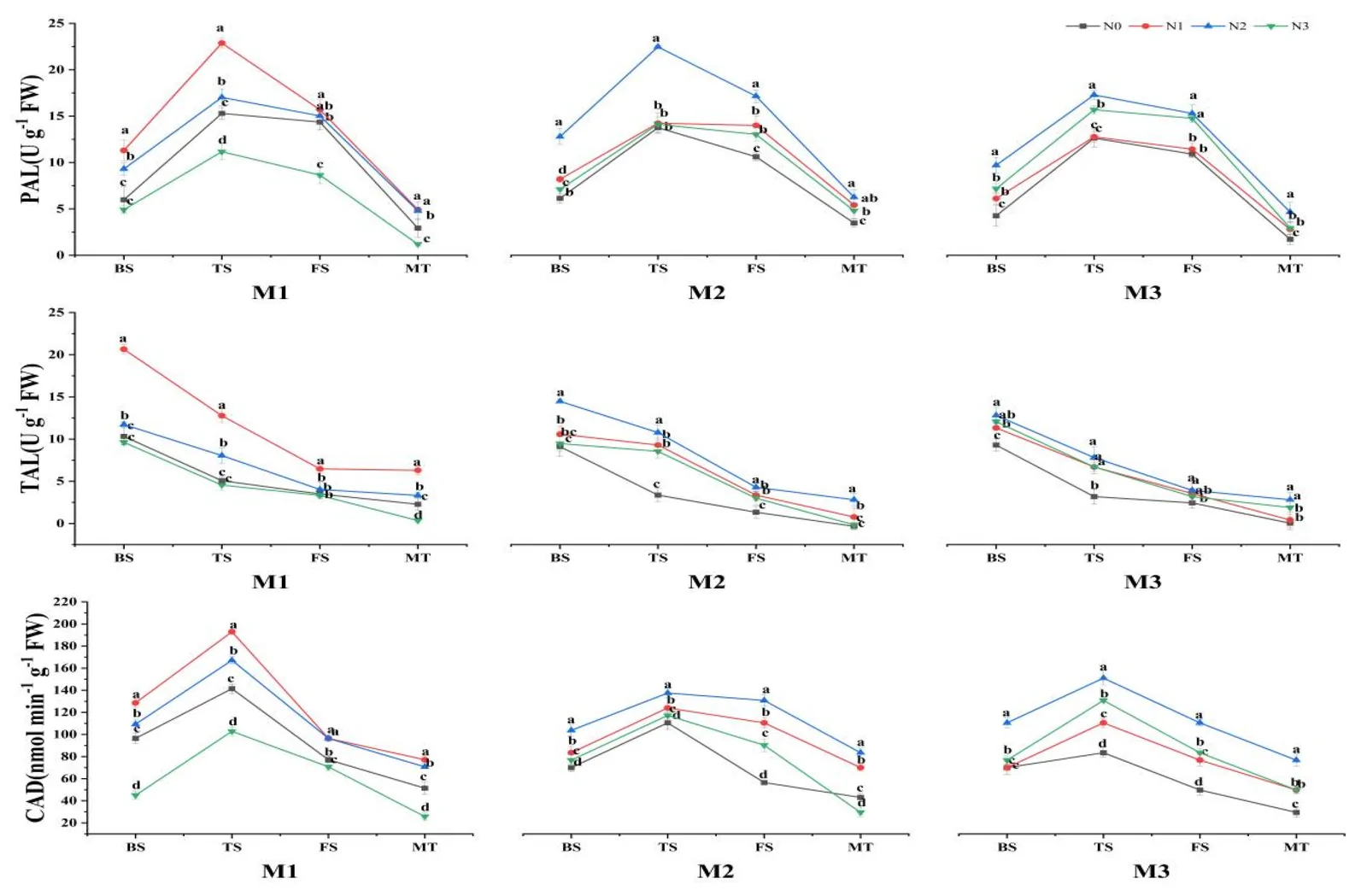
Effects of nitrogen-planting density interaction on maize stalk enzymatic activities. Note: The effect on the activity of enzymes related to the synthesis of lignin in the third internode of maize (Phenylalanine Ammonia-lyase (PAL), Tyrosine Ammonia-lyase (TAL), Cinnamyl Alcohol Dehydrogenase (CAD)). M1, M2, and M3 represent planting densities of 60,000, 75,000, and 90,000 plants ha^−1^, respectively; N0, N1, N2, and N3 represent nitrogen application rates of 0, 90, 180, and 270 kg ha^−1^, respectively; BS represents the Big trumpet stage, TS represents the Tasseling stage, FS represents the Filling stage, and MT represents the Maturity stage. a, b, c, and d indicate homogeneous groupings based on the results of the ANOVA. Error bar: represents the standard error.

The Phenylalanine Ammonia-Lyase (PAL) activity in the stalk initially increased and then decreased as the growth period progressed, reaching its peak at the tasseling stage (Figure 3. PAL). Under the same nitrogen application rate, the PAL activity decreased with increasing planting density. At densities M2 and M3, the activity decreased by 8.41% and 8.90%, respectively, compared to M1 (CK). Under the same planting density, the PAL activity initially increased and then decreased with increasing nitrogen fertilizer application. For M1 density, the PAL activity followed the order N1 > N2 > N0 > N3. The mean PAL activity under the M1N1 treatment was 13.70 U g^−1^, which is 4.06 U g^−1^, 2.16 U g^−1^, and 7.22 U g^−1^ higher than that under the M1N0, M1N2, and M1N3 treatments, respectively. The mean PAL activity under the M2N2 treatment was 13.63 U g^−1^, which was 6.78 U g^−1^, 4.62 U g^−1^, and 5.39 U g^−1^ higher than that under the M2N0, M2N1, and M2N3 treatments, respectively. At the M3 density, the PAL activity followed the order N2 > N3 > N1 > N0. The mean PAL activity under the M3N2 treatment was 11.74 U g^−1^, which was 4.36 U g^−1^, 3.46 U g^−1^, and 1.60 U g^−1^ higher than that under the M3N0, M3N1, and M3N3 treatments, respectively. (Data were averaged over four growth stages).

#### 3.5.2. Effect of Nitrogen-Planting Density Interaction on Tyrosine Ammonia-Lyase Activity

It can be observed that the activity of Tyrosine Ammonia-Lyase (TAL) exhibited a declining trend from the big trumpet stage to the maturity stage (Figure 3. TAL). Under the same nitrogen application rate, the TAL activity decreased with increasing planting density, with reductions of 4.21% and 6.39% at densities M2 and M3, respectively, compared to the control (M1). Under the same planting density, the TAL activity initially increased and then decreased with increasing nitrogen fertilizer application. For M1 densities, the TAL activity followed the order N1 > N2 > N0 > N3; for M2 density, the order was N2 > N1 > N3 > N0, while for M3 density, the order was N2 > N3 > N1 > N0. Compared to the N0 level, N1, N2, and N3 increased TAL activity by 3.47 U g^−1^, 2.96 U g^−1^, and 1.01 U g^−1^. (Average values across four growth stages).

#### 3.5.3. Effect of Nitrogen-Planting Density Interaction on Cinnamyl Alcohol Dehydrogenase Activity

Cinnamyl alcohol dehydrogenase (CAD) activity in maize increased initially and then decreased during the growth period, peaking at the tasseling stage (Figure 3). At a given nitrogen rate, CAD activity decreased with increasing planting density. Compared to the control (M1), the activity at M2 and M3 was reduced by 0.83% and 7.47%, respectively.

At a given planting density, CAD activity also showed an initial increase followed by a decrease with increasing nitrogen application. The ranking of CAD activity under different nitrogen treatments varied: it was N1 > N2 > N0 > N3 for M1; N2 > N1 > N3 > N0 for M2; and N2 > N3 > N1 > N0 for M3.

For the M1 density, compared to N0, the N1, N2, and N3 treatments increased CAD activity by 25.18, 37.51, and 1.07 nmol min^−1^ g^−1^, respectively. (Values are averages across the four growth stages).

### 3.6. Correlation Analysis Between Agronomic Traits, Mechanical Properties, Enzyme Activities, and Yield of Maize Stalks During the Tasseling Stage

**Figure 4:**
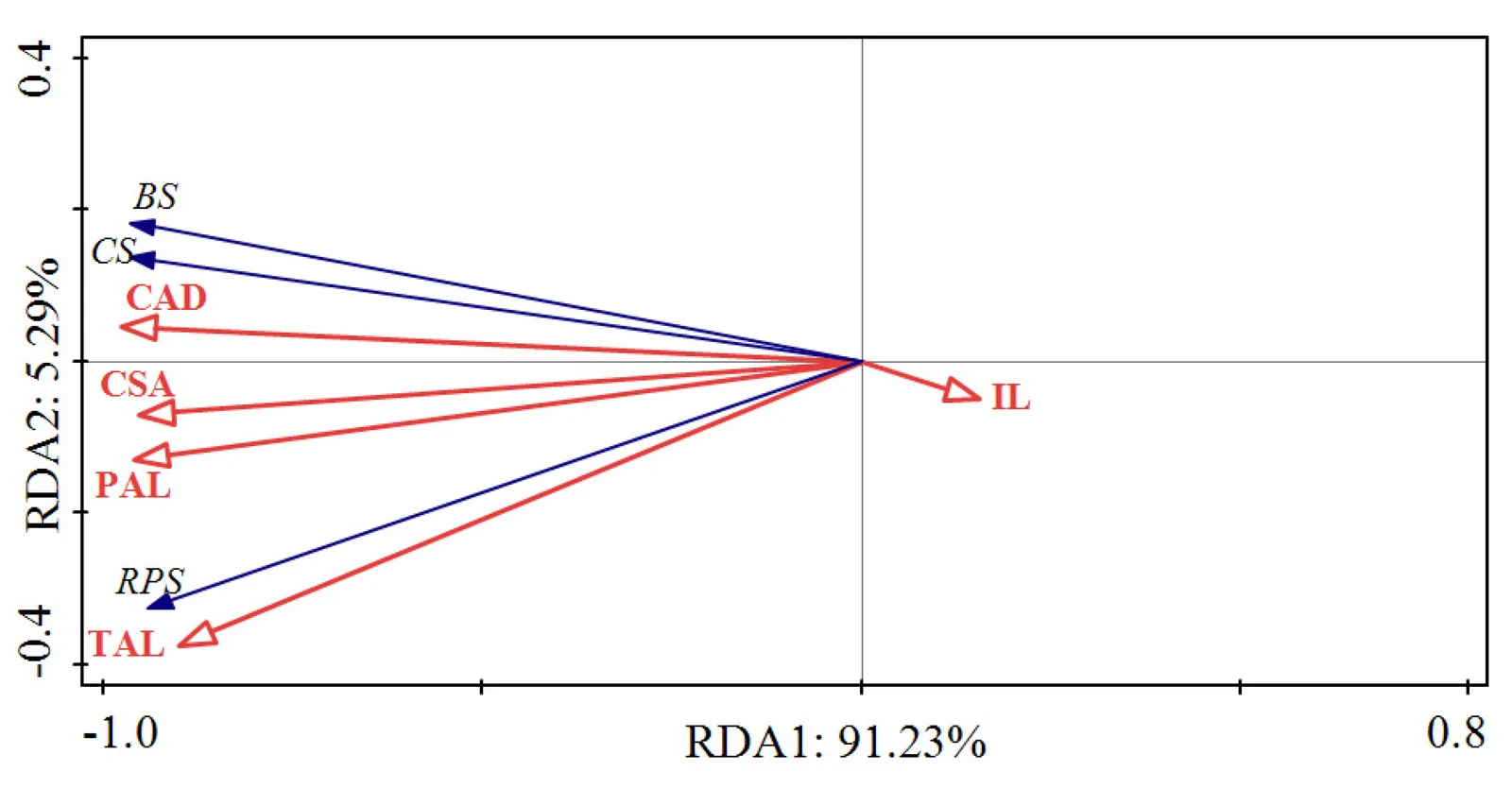
Correlation analysis of internode traits, enzymatic activities, and mechanical properties in maize stalks. Note: Redundancy analysis was used to identify the relationships between internode traits (red arrows), internode physiological characteristics (red arrows), and internode mechanical characteristics (blue arrows) at the third basal internode of maize plants during the tasseling stage. RDA, Redundancy Analysis; RPS, rind puncture strength; CS, compressive strength; BS, bending strength; IL, internode length; CSA, cross-sectional area; PAL, Phenylalanine Ammonia-Lyase; TAL, Tyrosine Ammonia-Lyase; CAD, Cinnamyl Alcohol Dehydrogenase.

Redundancy analysis was used to examine the relationship between basal internode characteristics and mechanical properties, as well as between enzyme activities and mechanical properties (Figure 4). The mechanical properties showed a negative correlation with internode length but a positive correlation with cross-sectional area. Conversely, these properties were positively associated with the measured enzyme activities. Collectively, the morphological traits and enzyme activities explained the variation in mechanical properties, with all evaluated indicators accounting for 96.5% of the total variation. Specifically, TAL and CAD enzyme activities were the key factors, explaining over 90% of the variation, followed by internode length, cross-sectional area, and PAL activity.

**Figure 5:**
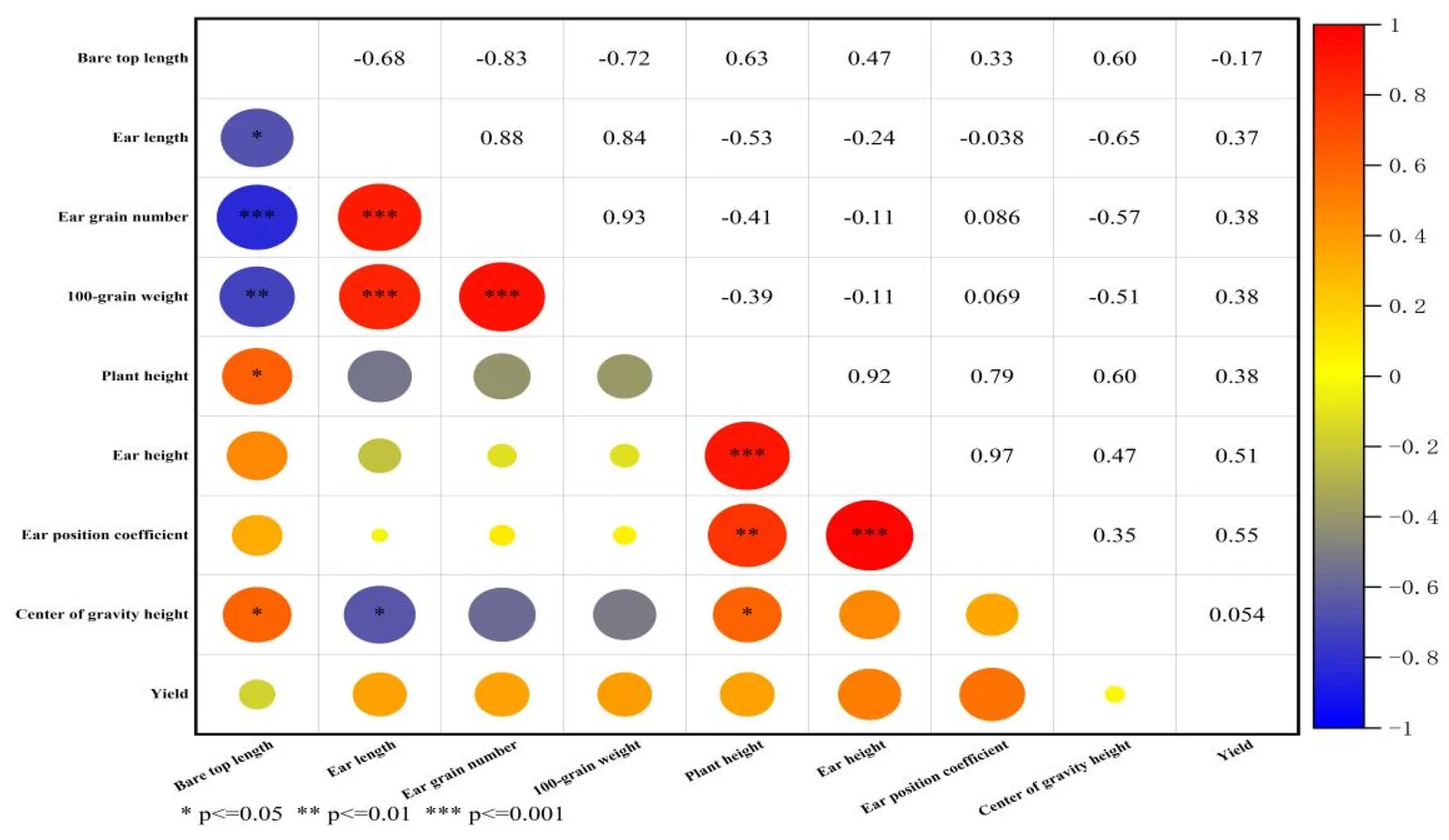
Correlation analysis of yield, yield components, and agronomic traits. Note: * represents a significant difference at *p* ≤ 0.05; ** represents a substantial difference at *p* ≤ 0.01; *** represents a significant difference at *p* ≤ 0.001.

As shown in Figure 5, the length of the bare top was inversely related to other yield components but positively related to stalk agronomic traits. Ear length was strongly positively correlated with both ear grain number and 100-grain weight (r = 0.88 and 0.84, respectively), and was also positively correlated with yield (r = 0.37). In contrast, it was negatively correlated with plant height, ear height, and ear height coefficient, and showed a significant negative correlation with the center of gravity height (r = −0.65). While both ear grain number and 100-grain weight were negatively correlated with stalk agronomic traits, they were highly positively correlated with each other (r = 0.93) and showed a positive correlation with yield (r = 0.38). Furthermore, all stalk agronomic traits were positively intercorrelated and were also positively correlated with yield.

## 4. Discussion

### 4.1. Effects of Nitrogen-Planting Density Interaction on Agronomic Traits and Stalk Mechanical and Physiological Characteristics of Maize Stalks

Increasing planting density could lead to a reduction in the cross-sectional area of maize stalks, while plant height, ear height, and center of gravity height increased. Applying nitrogen fertilizer can help increase stalk thickness, but it may also further enhance plant height, ear height, and center of gravity height. This result was consistent with the research conclusions of Tong [25], which jointly confirmed the interactive effect of planting density and nitrogen fertilizer application on the morphological construction of maize stalks. Qiao et al. [26] proposed that reasonably allocating planting density, constructing an efficient population structure, and coordinating contradictions between individuals and populations were key ways to increase maize yield. This study further revealed from the perspective of stalk traits that this allocation needed to take into account the balance of the mechanical properties of the stalks. Shi et al. [27] reported that under high-density planting conditions, increasing nitrogen fertilizer application can enhance maize stalk thickness, puncture strength, and bending performance, thereby reducing lodging rates. This study further found that the mechanical properties of maize stalks initially increased and then decreased with increasing nitrogen application rates, indicating that an appropriate nitrogen fertilizer could alleviate the decline in mechanical performance caused by high density, but excessive application had a negative impact. This discovery supplemented the details of the dose effect of nitrogen-planting density interaction on the regulation of the mechanical characteristics of stalks. Zhan et al. [21] found that the length of basal internodes first entered a rapid elongation period, followed by an increase in the cross-sectional area of internodes, and finally, the accumulation of photosynthetic products. Xu et al. [28] and Xie et al. [29] noted that insufficient or excessive nitrogen fertilizer application can lead to nitrogen stress throughout the maize growth period, affecting the accumulation of photosynthetic products in the later stages and thus reducing yield.

In this study, the activities of phenylalanine ammonia-lyase (PAL), tyrosine ammonia-lyase (TAL), and cinnamyl alcohol dehydrogenase (CAD) were significantly positively correlated with the mechanical strength of the stalks, which was consistent with the conclusions of Wang et al. [30]. The studies of Fang et al. [31] and Wang et al. [11] clearly demonstrated the key role of these enzymes in enhancing plant mechanical strength. This study further observed that the activities of PAL, TAL, and CAD decreased with increasing density; showed a trend of first increasing and then decreasing with nitrogen application; and exhibited significant dynamic changes during the growth period: the activities of PAL and CAD began to grow at the big trumpet stage, reached a peak at the tassel stage, and then decreased during the grain filling and maturity stages, and the activity of TAL was highest at the big trumpet stage and gradually decreased thereafter. This trend in enzyme activity reflects the shift in metabolic focus during the transition from vegetative growth to reproductive growth in plants, revealing the complexity of biochemical processes within maize plants and providing more concrete biochemical evidence for understanding the physiological regulation mechanism of stalk mechanical properties.

In this study, as density increased, the stalks became thinner, mechanical strength decreased, and lignin synthesis-related enzyme activity was downregulated, which was consistent with the research of Zheng et al. [8] and Li et al. [32], jointly elucidating the morphological and physiological synergistic mechanism of lodging risk induced by increased density. However, applying an appropriate amount of nitrogen could increase stalk diameter, hard skin puncture strength, compressive strength, and bending strength, and enhance lignin synthesis-related enzyme activity, thereby reducing the occurrence of lodging. Zhai et al. [22] proposed that increasing nitrogen fertilizer input could stimulate the growth of high-quality maize populations, significantly improve early stalk lodging resistance, delay stem senescence, enhance late stalk strength, and affect stalk composition. Zhao et al. [33] noted that the structural components (such as cellulose, hemicellulose, and lignin) and non-structural components (including soluble sugars and water) of the stalk had a significant impact on its mechanical and lodging resistance properties. However, when the nitrogen application rate reached 270 kg ha^−1^, the mechanical properties of the stalks decreased, the risk of lodging increased, and the yield decreased. It was speculated that this was related to excessive nitrogen causing plant overgrowth, internode elongation, and reduced material enrichment. In summary, appropriate density and nitrogen fertilizer application could provide guarantees for stable and high yields by coordinating stem morphological structure, mechanical properties, and physiological characteristics, while reducing mechanical grain harvesting energy consumption and increasing planting benefits. This conclusion underscores the practical value of nitrogen density optimization in enhancing the synergistic effect of maize lodging resistance and high yield, as informed by an integration of multidimensional research evidence.

### 4.2. Effects of Nitrogen-Planting Density Interaction on Maize Yield

In this study, yield increased with the increase in planting density, and the exposed length of the upper part of the maize ear significantly extended, which was consistent with the results of Jin et al. [34] and supported the population structure basis of yield increase under dense planting from the perspective of ear morphology. Maize yield was influenced by genotypic, environmental, and management factors [35], among which reasonably increasing planting density was a key approach to increase the number of effective ears per unit area and thus achieve yield increase [36,37,38]. Excessive nitrogen application could lead to a trend of first increase and then decrease in yield and related ear traits such as ear grain number and 100-grain weight, which was consistent with the reports of Li et al. [39] and Shao et al. [40], further verifying the threshold value of the yield effect of nitrogen fertilizer application. In this study, although the 100-seed weight of M1 and M3 was higher than that of M2, the number of grains per spike of M2 was higher than that of M1 and M3. The increase in effective grain number compensated for the negative impact of high planting density, and combined with the enhancement effect of appropriate nitrogen fertilization on stalk lodging resistance, the yield was ultimately improved. Although the maximum values of mechanical properties and enzyme activity were observed under the M1N2 configuration, the comprehensive yield index indicated that the M2N2 configuration was the best choice for achieving high and stable yields. This finding provided a basis for decision-making in optimizing nitrogen-dense combinations, combining physiological mechanisms with yield performance.

Reasonable close planting was a crucial measure to enhance maize yield and economic benefits, and optimizing the fertilization structure and the amount of fertilizer applied was key to increasing maize yield per unit area [41]. Some scholars suggested that the maximum yield application rate of nitrogen fertilizer for maize should be at least 300 kg ha^−1^. However, this study showed that increasing the planting density from 60,000 to 75,000 plants ha^−1^ could achieve high yield at a nitrogen fertilizer level of 180 kg ha^−1^ (M2N2), indicating that it was feasible to achieve high yield by appropriately increasing planting density and reducing nitrogen fertilizer application (from 270 kg ha^−1^ to 180 kg ha^−1^). It is worth noting that various factors, including variety and resource conditions, contributed to the high yield of maize through close planting. The increase in density needed to be controlled within a reasonable range to avoid problems such as lodging and other population structure imbalances. This conclusion clarified the feasible path for yield optimization under nitrogen-planting density interaction based on the integration of existing research on close planting and nitrogen fertilization. It provided a practical reference for resource-efficient maize production.

## 5. Conclusions

The responses of maize stalk mechanical properties, enzyme activities, and grain yield to nitrogen application rates varied significantly under different planting densities. High planting density altered the plant’s morphological structure and reduced the mechanical strength of the internodes. In contrast, appropriate nitrogen application improved plant morphology, as evidenced by increased internode diameter, and enhanced the activities of Phenylalanine Ammonia-lyase (PAL), Tyrosine Ammonia-lyase (TAL), and Cinnamyl Alcohol Dehydrogenase (CAD), thereby strengthening the mechanical properties of the stalk and ultimately increasing grain yield. In this study, based on the effects of mechanical properties and enzyme activity on yield, the M2N2 treatment demonstrated outstanding performance. In areas with similar conditions, the results of this study are expected to provide preliminary theoretical references for optimizing the management of dryland agricultural zones and offer a feasible approach for exploring high-yield, high-efficiency maize cultivation techniques. With further refinement and validation, this study may provide a reference direction for future improvements in related agronomic practices.

## Figures and Tables

**Table 1:** 2023–2024 corn growth stage table.

	Sowing	Fertilization	Big Trumpet Stage	Tasseling Stage	Filling Stage	Maturity Stage	Harvest
2023 year	24 April	4 June	2 July	20 July	19 August	22 September	22 September
2024 year	25 April	6 June	4 July	20 July	15 August	23 September	23 September

**Table 2:** Effect of nitrogen-planting density interaction on agronomic traits of maize stalks.

Planting Density	N Application Rate	Plant Height (cm)	Ear Height (cm)	Ear Height Coefficient (%)	Center of Gravity Height (cm)
M1	N0	247.17 ± 1.18 c	73.67 ± 1.89 c	29.80 ± 0.07 a	78.92 ± 1.77 ab
	N1	248.83 ± 2.59 bc	76.67 ± 1.46 b	30.81 ± 0.26 a	81.33 ± 0.94 ab
	N2	249.17 ± 0.71 a	76.50 ± 1.41 b	30.70 ± 0.62 a	80.33 ± 0.47 b
	N3	251.50 ± 1.65 ab	78.17 ± 0.24 a	31.08 ± 0.28 a	82.83 ± 0.71 a
M2	N0	249.83 ± 2.59 b	75.33 ± 0.47 b	30.15 ± 0.50 c	79.75 ± 1.53 b
	N1	250.50 ± 3.06 b	77.50 ± 2.12 ab	30.94 ± 0.46 b	83.08 ± 0.59 b
	N2	252.67 ± 2.36 ab	80.50 ± 0.71 a	31.86 ± 0.71 a	83.00 ± 0.47 b
	N3	260.17 ± 2.59 a	82.83 ± 1.65 a	31.84 ± 0.69 a	83.42 ± 1.30 a
M3	N0	253.67 ± 2.83 b	76.00 ± 1.41 c	29.96 ± 0.23 c	81.00 ± 0.47 b
	N1	256.50 ± 0.71 b	82.00 ± 2.40 ab	31.97 ± 0.88 b	84.17 ± 0.94 a
	N2	254.33 ± 2.83 b	81.67 ± 2.83 b	32.11 ± 0.76 b	85.00 ± 0.47 a
	N3	263.50 ± 1.18 a	86.33 ± 2.36 a	32.76 ± 0.75 a	80.92 ± 0.82 b
Planting density (M)		**	**	**	**
N application rate (N)		**	**	**	**
M × N		*	ns	ns	**

Note: M1, M2, and M3 represent planting densities of 60,000, 75,000, and 90,000 plants ha^−1^, respectively; N0, N1, N2, and N3 represent nitrogen application rates of 0, 90, 180, and 270 kg ha^−1^, respectively. Within the same column, values followed by different lowercase letters indicate significant differences at the 5% probability level among nitrogen fertilizer treatments under the same planting density. The standard deviation (SD) values are indicated by ±, with the larger SD value denoting the high heterogeneity of the characterization samples and the divergence of the individual values. Smaller SD values showed good homogeneity of the samples, and the data were clustered in a spike state. *, ** denote that the variable effects reach significant levels of 0.05 and 0.01, respectively, while “ns” indicates that the effect is not significant.

**Table 3:** Effect of nitrogen-planting density interaction on internode cross-sectional area and length of maize stalks at the tasseling stage.

Planting Density	N Application Rate	Cross Sectional Area (mm^2^)			Internode Length (cm)		
		3rd	4th	5th	3rd	4th	5th
M1	N0	655.58 ± 9.37 c	638.10 ± 8.02 c	570.32 ± 3.21 c	9.75 ± 0.26 ab	12.33 ± 0.33 ab	14.83 ± 0.94 a
	N1	801.22 ± 8.08 a	739.65 ± 7.08 a	658.72 ± 9.77 a	9.50 ± 0.09 b	11.83 ± 0.25 b	13.92 ± 0.35 b
	N2	747.52 ± 6.53 b	704.97 ± 8.07 b	621.68 ± 8.88 b	10.25 ± 0.54 a	12.33 ± 0.34 ab	14.08 ± 0.12 ab
	N3	587.03 ± 9.36 d	554.98 ± 9.04 d	491.68 ± 8.06 d	10.25 ± 0.82 a	13.08 ± 0.51 a	14.33 ± 0.71 ab
M2	N0	610.71 ± 7.04 c	567.69 ± 9.78 d	497.92 ± 10.23 d	10.75 ± 0.21 b	13.17 ± 0.24 a	15.42 ± 0.37 b
	N1	658.36 ± 7.81 b	612.73 ± 3.01 b	564.74 ± 3.93 b	11.42 ± 0.54 a	13.58 ± 0.14 a	16.00 ± 0.24 a
	N2	729.21 ± 8.83 a	691.90 ± 5.79 a	657.92 ± 6.66 a	9.75 ± 0.16 c	12.00 ± 0.61 b	14.00 ± 0.47 c
	N3	650.23 ± 6.65 b	594.09 ± 9.32 c	529.30 ± 3.83 c	9.50 ± 0.12 c	12.00 ± 0.47 b	14.33 ± 0.51 c
M3	N0	623.02 ± 6.81 d	582.59 ± 4.10 b	521.66 ± 2.36 c	12.58 ± 0.54 a	14.33 ± 0.38 a	15.92 ± 0.02 b
	N1	648.62 ± 4.64 c	586.30 ± 7.66 b	555.71 ± 5.26 b	11.67 ± 0.33 b	14.33 ± 0.33 a	16.17 ± 0.20 a
	N2	693.44 ± 9.24 a	648.58 ± 6.61 a	594.25 ± 7.31 a	9.50 ± 0.24 d	12.17 ± 0.24 b	14.33 ± 0.24 c
	N3	679.01 ± 8.85 b	642.04 ± 6.13 a	557.14 ± 10.43 b	11.17 ± 0.29 c	14.50 ± 0.66 a	15.92 ± 0.12 b
Planting density (M)		**	**	**	**	**	**
N application rate (N)		**	**	**	**	*	ns
M × N		**	**	**	**	**	**

Note: M1, M2, and M3 represent planting densities of 60,000, 75,000, and 90,000 plants ha^−1^, respectively; N0, N1, N2, and N3 represent nitrogen application rates of 0, 90, 180, and 270 kg ha^−1^, respectively. Within the same column, values followed by different lowercase letters indicate significant differences at the 5% probability level among nitrogen fertilizer treatments under the same planting density. The standard deviation (SD) values are indicated by ±, with the larger SD value denoting the high heterogeneity of the characterization samples and the divergence of the individual values. Smaller SD values showed good homogeneity of the samples, and the data were clustered in a spike state. *, ** denote that the variable effects reach significant levels of 0.05 and 0.01, respectively, while “ns” indicates that the effect is not significant.

**Table 4:** Effects of nitrogen-planting density interaction on maize yield and its composition.

Planting Density	N Application Rate	Ear Length (cm)	Bare Top Length (cm)	Ear Grain Number (Count)	100-Grain Weight (g)	Yield (Mg ha^−1^)
M1	N0	20.42 ± 0.1 a	1.14 ± 0.18 b	638.85 ± 9.97 c	45.20 ± 0.57 b	11.01 ± 0.30 a
	N1	20.52 ± 0.52 a	0.90 ± 0.18 b	691.55 ± 16.33 a	46.23 ± 0.11 a	11.09 ± 0.37 a
	N2	20.33 ± 0.03 a	0.96 ± 0.12 b	690.80 ± 12.45 b	44.53 ± 0.53 c	11.08 ± 0.42 a
	N3	19.69 ± 0.20 b	1.75 ± 0.21 a	610.90 ± 0.28 d	43.93 ± 0.46 c	10.39 ± 0.47 b
M2	N0	19.32 ± 0.01 c	1.48 ± 0.13 b	618.30 ± 12.02 d	43.70 ± 0.35 c	11.76 ± 0.30 d
	N1	20.53 ± 0.03 a	1.39 ± 0.15 b	705.00 ± 7.92 b	45.55 ± 0.35 a	13.15 ± 0.27 b
	N2	20.62 ± 0.11 a	0.99 ± 0.09 c	719.90 ± 2.55 a	45.60 ± 0.25 a	13.55 ± 0.17 a
	N3	19.78 ± 0.24 b	1.74 ± 0.12 a	676.50 ± 7.64 c	44.10 ± 0.21 b	12.25 ± 0.35 c
M3	N0	19.21 ± 0.43 b	1.61 ± 0.01 a	631.10 ± 19.23 c	44.18 ± 0.39 b	10.84 ± 0.41 b
	N1	20.48 ± 0.15 a	1.39 ± 0.16 b	657.05 ± 8.41 b	44.65 ± 0.64 ab	11.08 ± 0.28 b
	N2	20.66 ± 0.13 a	1.17 ± 0.17 c	692.05 ± 7.71 a	45.50 ± 0.40 a	11.92 ± 0.20 a
	N3	20.51 ± 0.18 a	1.47 ± 0.01 ab	685.90 ± 3.11 a	45.20 ± 0.21 ab	11.87 ± 0.31 a
Planting density (M)		**	**	**	**	**
N application rate (N)		**	**	**	**	**
M × N		**	**	**	*	**

Note: M1, M2, and M3 represent planting densities of 60,000, 75,000, and 90,000 plants ha^−1,^ respectively; N0, N1, and N2, N3 represent nitrogen application rates of 0, 90, 180, and 270 kg ha^−1^, respectively. Within the same column, values followed by different lowercase letters indicate significant differences at the 5% probability level among nitrogen fertilizer treatments under the same planting density. The standard deviation (SD) values are indicated by ±, with the larger SD value denoting the high heterogeneity of the characterization samples and the divergence of the individual values. Smaller SD values showed good homogeneity of the samples, and the data were clustered in a spike state. *, ** denote that the variable effects reach significant levels of 0.05 and 0.01, respectively.

## Data Availability

The original contributions presented in this study are included in the article. For further inquiries, please contact the corresponding authors.

## References

[1] Song Y., Zhang H., Meng F., Fan W., Wang N., Li Y., Li C. (2021). Effect of Nitrogen and Density Interaction on Agronomic Traits and Yield of Maize Cultivar Heyu 187. Heilongjiang Agric. Sci..

[2] Luo N., Wang X.Y., Hou J.M., Wang Y.Y., Wang P., Meng Q.F. (2020). Agronomic optimal plant density for yield improvement in the major maize regions of China. Crop Sci..

[3] Noor A.M. (2019). Dry Matter and Nitrogen Partitioning for High-Yielding Maize Hybrids Under Different Planting Densities and Nitrogen Rates and Their Interplay. Ph.D. Thesis.

[4] Li S., Wang K., Xie R., Hou PMing B., Yang X., Han D., Wang Y. (2016). Implementing Higher Population and Full Mechanization Technologies to Achieve High Yield and High Efficiency in Maize Production. Crops.

[5] Liu J., Wang Y., Niu J., Zhang Z., Niu Z., Zhu Q., Ye Y., Huang Y. (2021). Effects of Densification on Corn Yield and Lodging Resistance Under Different Nitrogen Levels. J. Agric..

[6] Deng Y., Wang C., Zhao L., Zhang L., Guo H., Wang L., Niu X., Wang M. (2017). Effects of Population Density on the Stem Traits and Soil Moisture in Maize and Their Correlation with Yield and Lodging Rate. Acta Agric. Boreali-Sin..

[7] Zheng L., Liu H., Li Z., Wang Y., Tian S., Li A. (2020). Effect of Different Planting Densities on Maize Growth Under the Condition of Water and Fertilizer Integration. J. Shanxi Agric. Sci..

[8] Zheng Y., Chen D., Wei P., Lu P., Yang J., Luo S., Ye K., Song B. (2021). Effects of planting density on lodging resistance and grain yield of spring maize stalks in Guizhou province. Acta Agron. Sin..

[9] Liu X., Ma X., Dou P., Huang K., Wang X., Zhang D., Kong F., Yuan J. (2017). Effect of planting density on stem characteristics and yield of summer maize in the Hilly Central Sichuan Basin, China. Chin. J. Eco-Agric..

[10] Huang H., Chang Y., Wu C., Hu W., Gu Y. (2014). Effects of population density on strength and related physiological indexes of maize stalk. J. Northwest A&F Univ. (Nat. Sci. Ed.).

[11] Wang T., Zhang L., Han Q., Zheng F., Wang T., Feng N., Wang T. (2015). Effects of stalk cell wall and tissue on the compressive strength of maize. Plant Sci. J..

[12] Liu Q., Luo L., Zheng L. (2018). Lignins: Biosynthesis and biological functions in plants. Int. J. Mol. Sci..

[13] Franke R., Hemm M.R., Denault J.W., Ruegger M.O., Humphreys J.M., Chapple C. (2002). Changes in secondary metabolism and deposition of an unusual lignin in the ref8 mutant of Arabidopsis. Plant J..

[14] Liu X., Jin J., He P., Gao W., Li W. (2007). Effect of Potassium Chloride on Lignin Metabolism and Its Relation to Resistance of Corn to Stalk Rot. Sci. Agric. Sin..

[15] Erisman J.W., Sutton M.A., Galloway J., Klimont Z., Winiwarter W. (2008). How a century of ammonia synthesis changed the world. Nat. Geosci..

[16] Lu P., Luo S., Ye K., Song B., Zhang J. (2020). Effects of Plant Density and Nitrogen Fertilizer on Maize Growth and Mechanically Harvesting Traits. J. Mt. Agric. Biol..

[17] Gao X., Gao J., Yu X., Wang Z., Sun J., Su Z., Hu S., Ye J., Wang H., Cui C. (2012). Stalks Lodging-Resistance Characteristics and Yield Traits among Different Maize Varieties under High Close Planting. J. Maize Sci..

[18] Zhai J., Xue J., Zhang Y., Zhang G., Shen D., Wang Q., Liu C., Li S. (2021). Effect of Nitrogen Application Rate on Lodging Resistance of Spring Maize Stalks under Integrated Irrigation with Water and Fertilizer. J. Maize Sci..

[19] Shi D. (2016). Research on Dense Planting Effects and Density Tolerance Mechanisms in High-Yielding Summer Maize and N-Fertilizer Regulation. Ph.D. Thesis.

[20] Liu X., Gu W., Li C., Li J., Wei S. (2021). Effects of nitrogen fertilizer and chemical regulation on spring maize lodging characteristics, grain filling, and yield formation under high planting density in Heilongjiang Province. China J. Integr. Agric..

[21] Zhan X., Kong F., Liu Q., Lan T., Liu Y., Xu J., Ou Q., Chen L., Kessel G., Kempenaar C. (2022). Maize basal internode development significantly affects stalk lodging resistance. Field Crops Res..

[22] Zhai J., Zhang Y., Zhang G., Tian M., Xie R., Ming B., Hou P., Wang K., Xue J., Li S. (2022). Effects of nitrogen fertilizer management on stalk lodging resistance traits in summer maize. Agriculture.

[23] Liu F., Zhou F., Wang X., Zhan X., Guo Z., Liu Q., Wei G., Lan T., Feng D., Kong F. (2023). Optimizing nitrogen management enhances stalk lodging resistance and grain yield in dense planting maize by improving canopy light distribution. Eur. J. Agron..

[24] Xie G., Liang M., Chen P., Zhang C., Fan M., Wang C., Zhao L. (2024). The effects of tillage and the combined application of organic and inorganic fertilizers on the antioxidant enzyme activity and yield of maize leaves. Agronomy.

[25] Tong T. (2020). Regulation Effects of Nitrogen Fertilizer and Density on Stem Lodging and Grain Filling of Maize. Master’s Thesis.

[26] Qiao J., Zhang P., Shao Y., Liu J., Li C., Zhang M., Huang L. (2022). Effects of Different Planting Densities and Varieties on Dry Matter Production and Yield Components of Summer Maize. Crops.

[27] Shi D., Li Y., Zhang J., Liu P., Zhao B., Dong S. (2016). Effects of plant density and nitrogen rate on lodging-related stalk traits of summer maize. Plant Soil Environ..

[28] Xu F., Zhao G., Tian Q., Chang X., Yang Y., Wang D., Liu X. (2012). Effects of nitrogen fertilization on grain yield and processing quality of different wheat genotypes. Plant Nutr. Fertil. Sci..

[29] Xie J., Han X., Wang L., Hou Y. (2013). Effect of Different Nitrogen Application Modes on Maize Yield, Nutrition Uptake and Utilization Efficiency. J. Maize Sci..

[30] Wang K., Zhao X., Yao X., Yao Y., Bai Y., Wu K. (2019). Relationship of stem characteristics and lignin synthesis with lodging resistance of hulless barley. Acta Agron. Sin..

[31] Fang X., Liu X., Zhang Y., Huang K., Zhang Y., Li Y., Nie J., She H., Ruan R., Yuan X. (2018). Effects of uniconazole or gibberellic acid application on the lignin metabolism in relation to lodging resistance of culm in common buckwheat (*Fagopyrum esculentum* M.). J. Agron. Crop Sci..

[32] Li B., Gao F., Ren B., Dong S., Liu P., Zhao B., Zhang J. (2021). Lignin metabolism regulates lodging resistance of maize hybrids under varying planting density. J. Integr. Agric..

[33] Zhao X., Zhou S. (2022). Research progress on traits and assessment methods of stalk lodging resistance in maize. Acta Agron. Sin..

[34] Jin R., Li Z., Yang Y., Zhou F., Du L., Li X., Kong F., Yuan J. (2020). Effects of density and row spacing on population light distribution and male and female spike differentiation of summer maize in the hilly area of central Sichuan. Acta Agron. Sin..

[35] Incognito S.J.P., Maddonni G.Á., López C.G. (2020). Genetic control of maize plant architecture traits under contrasting plant densities. Euphytica.

[36] Wang K., Wang K., Wang Y., Zhao J., Zhao R., Wang X., Li J., Liang M., Li S. (2012). Effects of Density on Maize Yield and Yield Components. Sci. Agric. Sin..

[37] Meng Q., Hou P., Wu L., Chen X., Cui Z., Zhang F. (2013). Understanding production potentials and yield gaps in intensive maize production in China. Field Crops Res..

[38] Hou P., Liu Y., Liu W., Liu G., Xie R., Wang K., Ming B., Wang Y., Zhao R., Zhang W. (2020). How to increase maize production without extra nitrogen input. Resour. Conserv. Recycl..

[39] Li C., Zheng H., Li Y., Li H. (2010). Effect of Planting Density on the Yield and Development of Maize Ear. Sci. Agric. Sin..

[40] Shao H., Wu X.B., Chi H.H., Zhu F.B., Liu J.H., Duan J.H., Shi W.J., Xu Y., Mi G.H. (2024). How does increasing planting density affect nitrogen use efficiency of maize: A global meta-analysis. Field Crops Res..

[41] Meng Z., Wang Y., Wang X., Shen D. (2011). The Effects of Increasing Density on Summer Grouting Characteristics and Yield. J. Henan Agric. Sci..

