# Correction: Chen et al. The Effect of the Interaction of Nitrogen Fertilization with Planting Density on Maize (*Zea mays* L.) Yield, Stalk Mechanical Properties, and Enzyme Activity. *Plants* 2026, *15*, 459

**DOI:** 10.3390/plants15142188

**Published:** 2026-07-17

**Authors:** Pei Chen, Li Zhao, Zhi-Long Zhang, Lin-Zhuan Song, Xue-Feng Zhao, Xin Zhang, Xin-Rong Duan, Min Liang, Chang Zhang, Chuang-Yun Wang

**Affiliations:** 1 College of Agriculture, Shanxi Agricultural University, Jinzhong 030801, China; 20233239@stu.sxau.edu.cn (P.C.); lizhao@sxau.edu.cn (L.Z.); 15947185502@163.com (Z.-L.Z.); 202430209@stu.sxau.edu.cn (L.-Z.S.); 202420172@stu.sxau.edu.cn (X.Z.); 202430127@stu.sxau.edu.cn (X.-R.D.); 20233243@stu.sxau.edu.cn (M.L.); 20232189@stu.sxau.edu.cn (C.Z.); 2 Yuci District Meteorological Bureau of Shanxi Province, Jinzhong 030600, China; ertou01@163.com

In the original publication [1], there was a mistake in Table 3 (Effect of nitrogen-planting density interaction on internode cross-sectional area and length of maize stalks at the tasseling stage) as published. Due to the authors’ oversight during the proofreading stage, some data within the table were misplaced. The corrected Table 3 appears below. The authors state that the scientific conclusions are unaffected. This correction was approved by the Academic Editor. The original publication has also been updated.

## Figures and Tables

**Table 3:** Effect of nitrogen-planting density interaction on internode cross-sectional area and length of maize stalks at the tasseling stage.

Planting Density	N Application Rate	Cross Sectional Area (mm^2^)			Internode Length (cm)		
		3rd	4th	5th	3rd	4th	5th
M1	N0	655.58 ± 9.37 c	638.10 ± 8.02 c	570.32 ± 3.21 c	9.75 ± 0.26 ab	12.33 ± 0.33 ab	14.83 ± 0.94 a
	N1	801.22 ± 8.08 a	739.65 ± 7.08 a	658.72 ± 9.77 a	9.50 ± 0.09 b	11.83 ± 0.25 b	13.92 ± 0.35 b
	N2	747.52 ± 6.53 b	704.97 ± 8.07 b	621.68 ± 8.88 b	10.25 ± 0.54 a	12.33 ± 0.34 ab	14.08 ± 0.12 ab
	N3	587.03 ± 9.36 d	554.98 ± 9.04 d	491.68 ± 8.06 d	10.25 ± 0.82 a	13.08 ± 0.51 a	14.33 ± 0.71 ab
M2	N0	610.71 ± 7.04 c	567.69 ± 9.78 d	497.92 ± 10.23 d	10.75 ± 0.21 b	13.17 ± 0.24 a	15.42 ± 0.37 b
	N1	658.36 ± 7.81 b	612.73 ± 3.01 b	564.74 ± 3.93 b	11.42 ± 0.54 a	13.58 ± 0.14 a	16.00 ± 0.24 a
	N2	729.21 ± 8.83 a	691.90 ± 5.79 a	657.92 ± 6.66 a	9.75 ± 0.16 c	12.00 ± 0.61 b	14.00 ± 0.47 c
	N3	650.23 ± 6.65 b	594.09 ± 9.32 c	529.30 ± 3.83 c	9.50 ± 0.12 c	12.00 ± 0.47 b	14.33 ± 0.51 c
M3	N0	623.02 ± 6.81 d	582.59 ± 4.10 b	521.66 ± 2.36 c	12.58 ± 0.54 a	14.33 ± 0.38 a	15.92 ± 0.02 b
	N1	648.62 ± 4.64 c	586.30 ± 7.66 b	555.71 ± 5.26 b	11.67 ± 0.33 b	14.33 ± 0.33 a	16.17 ± 0.20 a
	N2	693.44 ± 9.24 a	648.58 ± 6.61 a	594.25 ± 7.31 a	9.50 ± 0.24 d	12.17 ± 0.24 b	14.33 ± 0.24 c
	N3	679.01 ± 8.85 b	642.04 ± 6.13 a	557.14 ± 10.43 b	11.17 ± 0.29 c	14.50 ± 0.66 a	15.92 ± 0.12 b
Planting density (M)		**	**	**	**	**	**
N application rate (N)		**	**	**	**	*	ns
M × N		**	**	**	**	**	**

Note: M1, M2, and M3 represent planting densities of 60,000, 75,000, and 90,000 plants ha^−1^, respectively; N0, N1, N2, and N3 represent nitrogen application rates of 0, 90, 180, and 270 kg ha^−1^, respectively. Within the same column, values followed by different lowercase letters indicate significant differences at the 5% probability level among nitrogen fertilizer treatments under the same planting density. The standard deviation (SD) values are indicated by ±, with the larger SD value denoting the high heterogeneity of the characterization samples and the divergence of the individual values. Smaller SD values showed good homogeneity of the samples, and the data were clustered in a spike state. *, ** denote that the variable effects reach significant levels of 0.05 and 0.01, respectively, while “ns” indicates that the effect is not significant.
